# Risk Factors Associated with Positive QuantiFERON-TB Gold In-Tube and Tuberculin Skin Tests Results in Zambia and South Africa

**DOI:** 10.1371/journal.pone.0018206

**Published:** 2011-04-04

**Authors:** Kwame Shanaube, James Hargreaves, Katherine Fielding, Ab Schaap, Katherine-Anne Lawrence, Bernadette Hensen, Charalambos Sismanidis, Angela Menezes, Nulda Beyers, Helen Ayles, Peter Godfrey-Faussett

**Affiliations:** 1 ZAMBART Project, University of Zambia, Lusaka, Zambia; 2 Department of Infectious Disease Epidemiology, London School of Hygiene and Tropical Medicine, London, United Kingdom; 3 Department of Clinical Research, London School of Hygiene and Tropical Medicine, London, United Kingdom; 4 Desmond Tutu TB Centre, Stellenbosch University, Stellenbosch, South Africa; 5 Stop TB Department, Monitoring and Evaluation Unit, World Health Organization, Geneva, Switzerland; McGill University, Canada

## Abstract

**Introduction:**

The utility of T-cell based interferon-gamma release assays for the diagnosis of latent tuberculosis infection remains unclear in settings with a high burden of tuberculosis.

**Objectives:**

To determine risk factors associated with positive QuantiFERON-TB Gold In-Tube (QFT-GIT) and tuberculin skin test (TST) results and the level of agreement between the tests; to explore the hypotheses that positivity in QFT-GIT is more related to recent infection and less affected by HIV than the TST.

**Methods:**

Adult household contacts of tuberculosis patients were invited to participate in a cross-sectional study across 24 communities in Zambia and South Africa. HIV, QFT-GIT and TST tests were done. A questionnaire was used to assess risk factors.

**Results:**

A total of 2,220 contacts were seen. 1,803 individuals had interpretable results for both tests, 1,147 (63.6%) were QFT-GIT positive while 725 (40.2%) were TST positive. Agreement between the tests was low (kappa = 0.24). QFT-GIT and TST results were associated with increasing age (adjusted OR [aOR] for each 10 year increase for QFT-GIT 1.15; 95% CI: 1.06–1.25, and for TST aOR: 1.10; 95% CI 1.01–1.20). HIV positivity was less common among those with positive results on QFT-GIT (aOR: 0.51; 95% CI: 0.39–0.67) and TST (aOR: 0.61; 95% CI: 0.46–0.82). Smear positivity of the index case was associated with QFT-GIT (aOR: 1.25; 95% CI: 0.90–1.74) and TST (aOR: 1.39; 95% CI: 0.98–1.98) results. We found little evidence in our data to support our hypotheses.

**Conclusion:**

QFT-GIT may not be more sensitive than the TST to detect risk factors associated with tuberculous infection. We found little evidence to support the hypotheses that positivity in QFT-GIT is more related to recent infection and less affected by HIV than the TST.

## Introduction

Tuberculosis continues to be a major public health problem in sub-Sahara Africa. The incidence of tuberculosis [1] is being accelerated by high rates of HIV co-infection [2], [3]. Targeted testing and treatment of latent tuberculosis infection (LTBI) especially among HIV positive individuals is an important strategy to reduce the incidence of tuberculosis [4].

Currently, LTBI detection relies on the tuberculin skin test (TST) in most countries with high incidence of tuberculosis although this is not routinely performed and is perceived as a barrier to accessing TB preventive therapy [5]. However, TST has many reported limitations. These include low specificity in populations with high levels of BCG vaccination or significant exposure to non-tuberculosis mycobacteria (NTM), and reduced sensitivity in immunocompromised individuals such as those with HIV infection [6], [7].

T-cell based interferon-gamma release assays (IGRAs) such as the QuantiFERON-TB Gold In-Tube (QFT-GIT) can now also be used to detect LTBI [8]. IGRAs are in-vitro blood tests based on interferon-γ release after T-cell stimulation by antigens more specific to *Mycobacterium tuberculosis (Mtb)* than the purified protein derivative used in TST. IGRAs are therefore designed to have high specificity that is unaffected by BCG vaccination and cross-reactivity with most NTM [8]. There is also some evidence of greater sensitivity among HIV positive individuals [9], [10] compared to the TST. Current literature suggests that IGRAs detect responses of effector T-cells that have recently encountered antigens *in vivo,* while TST reflects the mobilization of a wider spectrum of memory T-cells that are long-lived [11].

The use of IGRAs in developed countries is rapidly expanding but their performance in settings with a high prevalence of tuberculosis and HIV still requires further research [12], [13]. There is growing evidence that IGRAs performance varies in different settings [14]. In high-TB burden settings, the results of IGRAs may be influenced by factors that affect the immune response [15] such as HIV co-infection, BCG vaccination, malnutrition, tropical infections and widespread exposure to NTM. Recent studies done in low and middle income settings [16] showed a large reduction in the proportion of positive test results for both QFT-GIT and TSPOT in HIV infected individuals. Another recent study in Bangladesh showed that malnutrition and helminth infections were associated with indeterminate QFT-GIT results in children [17].

Significant challenges exist in directly assessing whether IGRAs are superior to TST in diagnosing LTBI as there remains no gold standard against which to compare either test. In the absence of a practical gold standard for *Mtb* infection, exposure to an infectious TB index case has been used as a surrogate measure of infection [18], [19], [20], [21]. Studies from low-TB burden countries indicate that the IGRAs correlate better, along a gradient of exposure, than the TST [14]. Nevertheless, in high-TB burden settings, the TST performs reasonably well and correlates as well, or better, with proxy measures of exposure [14]. There are limited data on the comparison of QFT-GIT and TST in relation to *Mtb* exposure as a surrogate measure of infection and the influence of age [19].

In this study, we describe the prevalence of tuberculous infection among household contacts of recently diagnosed tuberculosis patients as measured by QFT-GIT and TST in 24 communities with a high prevalence of TB and HIV in Zambia and South Africa. We also determine risk factors associated with positive QFT-GIT and TST results and the level of agreement between the tests in each community. We use data from two recent TST surveys [22] to explore the correlation between community TB transmission and infection prevalence in contacts as measured by QFT-GIT and TST. A TST survey, if conducted correctly and technically interpretable, allows an estimation of the extent of *Mtb* transmission that has occurred in the community [23].

Finally, we formally assess the extent to which our results are compatible with expected findings on the basis of a number of prior hypotheses about the characteristics of each test. Previous studies have given rise to prevailing views about the expected performance of both TST and IGRAs [7], [11], [14], [16]. We therefore explore whether our data support the hypotheses that positivity in QFT-GIT is more related to recent infection and less affected by HIV than the TST.

## Methods

### Ethics statement

Ethics approval for the study was obtained from the research ethics committees of the University of Zambia, the London School of Hygiene and Tropical Medicine and Stellenbosch University. All individuals involved in the study gave written informed consent.

### Study setting

This cross-sectional study was nested within a large community randomized trial of interventions to reduce tuberculosis transmission, the Zambia South Africa TB and AIDS Reduction Study, (ZAMSTAR) in 24 selected communities in Zambia and South Africa [24]. We defined a “community” as the population (minimum size of 25,000) accessing one tuberculosis diagnostic centre and this was the unit of randomization for the ZAMSTAR trial. The communities selected were in five provinces of Zambia (16 communities) and in Western Cape Province of South Africa (8 communities) and included both urban and rural communities. The design of the ZAMSTAR study is described elsewhere [24], [25].

Baseline measurement of tuberculous infection in all ZAMSTAR communities was estimated by means of TST surveys among primary school children [22]. These community-wide surveys served three objectives: to characterize ZAMSTAR communities, with regards to TB infection, in relative terms; to inform the randomization of the communities into intervention arms; and to provide data for one of ZAMSTAR's secondary outcomes.

Zambia and South Africa have among the highest tuberculosis incidence [26] and HIV seroprevalence rates [27] in Africa and globally. The estimated HIV prevalence in new tuberculosis cases is 70% [26].

### Participants

From April 2007 to July 2008 we recruited newly notified adult tuberculosis cases from the 24 ZAMSTAR communities, subsequently referred to as index cases, to the study. All tuberculosis cases (pulmonary smear positive, smear negative or extrapulmonary) were eligible if recruited within a month after being notified in the tuberculosis register and started on treatment at a government clinic. We obtained written informed consent from those accepting to take part in the study. In addition, we sought permission from these index cases to visit their households where we invited household members to participate. We made at least three attempts to visit household members who were absent during the first visit to the household.

We defined household contacts as individuals at least 15 years old, who generally slept in the home, ate with the index case and who identified a common household head. We asked all household contacts for individual signed consent before participating in the study. This study focuses on this population of household contacts of newly diagnosed tuberculosis cases.

### Measures

Consenting household contacts had blood drawn for HIV antibodies and QFT-GIT testing. Tuberculin skin tests were also performed. A standardized questionnaire was administered to all contacts by trained interviewers, who collected information on risk factors associated with tuberculous infection. Sputum microscopy for index cases was performed as part of the clinic routine services and the results were recorded in the TB registers.

HIV testing was done using the Abbot Murex HIV Ag/Ab combination ELISA (Murex Biotech, Dartford, United Kingdom). All individuals were encouraged to attend counseling and HIV testing at the local health centre. In South Africa, HIV positive individuals were advised to go for TB preventive therapy in accordance with National Tuberculosis Control Program guidelines [28] while in Zambia this is not yet government policy. However, in Zambia, preventive therapy was offered to eligible contacts through collaboration with another study operating in the ZAMSTAR sites.

#### QFT-GIT procedure

QFT-GIT test was performed according to the manufacturer's instructions [29]. For four Zambian and all the South African communities, QFT-GIT processing was done centrally at our research laboratories in Lusaka and Stellenbosch University Medical School respectively. However, for twelve Zambian remote communities, QFT-GIT processing was decentralized. In these communities, blood samples were collected, incubated, separated and stored locally. Tubes were incubated for 16–24 hours at 37°C and plasma was harvested and frozen at –20°C. Frozen samples from these sites were transported monthly to the central laboratory in Lusaka where the ELISA was performed manually in batches.

#### TST procedure

The skin testing was conducted using 2 TU (Tuberculin Units) of PPD RT23 with Tween, supplied by the Statens Serum Institut (Copenhagen, Denmark). All tests were administered and read by nurses who were trained according to the standard IUATLD protocol [23]. A dose of 0.1 ml was injected intradermally on the left forearm. Skin reactions were read using calipers 72 hours later. A positive TST was defined as an induration of ≥10 mm. Blood for QFT-GIT was drawn before TST was administered usually on the same day.

### Statistical analysis

Data were double entered into a “Microsoft SQL Server” database and checked for errors. Analysis was performed using STATA (version 11.0).

The characteristics of the study population were described using frequencies and percentages for categorical variables and the median and interquartile range for quantitative variables. *Prevalence of infection* was defined as the number of QFT-GIT or TST positive results divided by the total number of individuals with interpretable (positive and negative) results. Individuals having missing TST or indeterminate QFT-GIT results were excluded from the analysis. These did not differ significantly from those that had interpretable results (results not shown). Furthermore, household contacts on TB treatment were excluded from analysis.

The level of agreement between test results was assessed for each community using Cohen's kappa coefficient. By convention, kappa values of less than 0.4 generally indicate poor agreement. Correlation between continuous interferon-γ values (IU/ml) and TST induration (mm) was assessed using Spearman's correlation coefficient for each community.

The distribution of positive reactions to each test in relation to established individual and household level risk factors for LTBI was described. The strength of relationship between risk factors and QFT-GIT/TST positivity was assessed using random effects logistic regression. The random effects approach specified the household of residence as the clustering variable. All models were adjusted for age, sex and community of residence. We present adjusted odds ratios (ORs) and 95% confidence intervals (CIs) for each risk factor.

Finally, we formally assessed four hypotheses related to the expected performance of the tests. We explored whether our data were compatible with expected findings on the basis of a number of prior hypotheses about the characteristics of each test. Ideally, we would have had a gold standard measure of LTBI against which to compare both tests. However, no such test currently exists. Furthermore, the natural history of LTBI remains a source of debate [11] and as in other studies of LTBI we had no direct measure of exposure to *Mtb* either recently or in the past. However, we did have data on three proxies related to prior exposure to *Mtb* (age) or recent exposure to *Mtb* (proximity to and infectivity of the index case).

Thus, we tested: (i) whether HIV infection was associated with a negative TST result, among those with a positive QFT-GIT, since we expect HIV infection to cause more false negative results with TST than QFT-GIT. We restricted the analysis to individuals with a positive QFT-GIT result and used random effects logistic regression models as described previously.

We then explored: (ii) whether age was more strongly associated with a positive TST result than with a positive QFT-GIT result since we expected TST to be more likely to detect evidence of lifetime infection with *Mtb* while QFT-GIT was more likely to detect recent infections; (iii) whether living with a smear positive index case was more strongly associated with a positive QFT-GIT result than a positive TST result, since we expected that QFT-GIT would be more strongly associated with recent infections than TST; and (iv), for the same reason as (iii), whether sleeping in the same room as the index case was more strongly associated with QFT-GIT than TST positivity.

For these final three hypotheses we conducted matched-pairs analysis using conditional logistic regression in an approach similar to that used by Ewer and others (21), where the outcome is the result of the TST or QFT-GIT test. We used Wald tests to assess the strength of evidence for an interaction between the test used and the factor of interest (age, living with a smear positive index case, sleeping in same room as index case). In these models we specified the individual identifier to indicate the paired data, and calculated confidence intervals using robust standard errors taking account of household level clustering.

## Results

**Figure 1:**
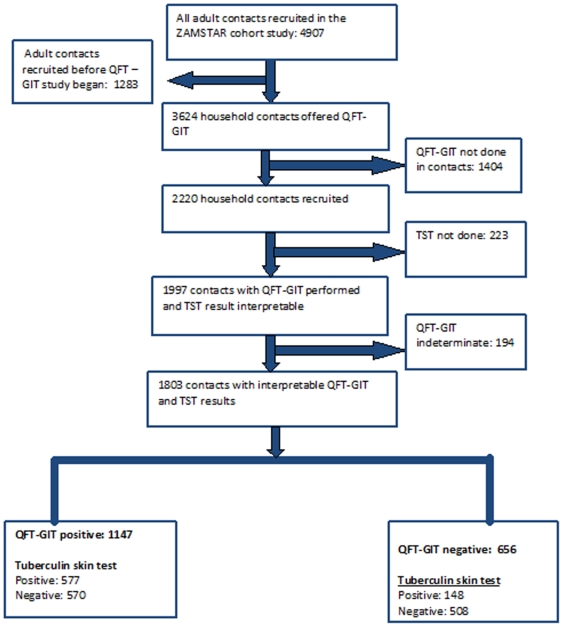
Flow diagram of study participants. QFT-GIT (QuantiFERON-TB Gold In Tube) not done was due to refusal (18.4%), being absent (18.3%), insufficient blood samples (0.4%) or missing data (1.6%). TST (Tuberculin skin test) not done was due to refusal (5.6%), not returning for reading (3.6%) or missing data (0.8%). Individuals with QFT-GIT/TST not done and those with indeterminate QFT-GIT results were excluded from analysis.

Figure 1 shows the flow chart of the study participants. A total of 2220 contacts were recruited across the 24 communities. As shown in table 1, the study population was predominantly women (69.9%), most had attended secondary education (52.1%) and had no history of smoking (81.9%) or alcohol consumption (75.2%). 765/2036 (37.6%) of household contacts were HIV positive, and 766/1507 (50.8%) lived with a smear positive TB index case.

**Table 1:** Characteristics of the study population.

	Study participants n (column %)
Total	2220
***Sex***	
Male	666 (30.1)
Female	1545 (69.9)
Missing	9
***Age group (years)***	
15–24	835 (38.3)
25–34	556 (25.5)
35–44	302 (13.9)
45–54	246 (11.3)
55–64	134 (6.1)
>65	106 (4.9)
Missing	41
Age in years: Median 28 (IQR:21–42); mean 33	
***Highest level of education***	
Not attended school	142 (6.5)
Primary school	689 (31.9)
Secondary school	1126 (52.1)
College or University	203 (9.4)
Missing	60
***Smoking habits***	
Daily smoker	220 (10.0)
Occasional smoker	73 (3.3)
Ex-smoker	104 (4.7)
Never smoked	1801 (81.9)
Missing	22
1 ***Alcohol consumption***	
No	1648 (75.2)
Yes	544 (24.8)
Missing	28
***Household size (adults)***	
1–3	331 (15.0)
4–6	848 (38.4)
7–9	554 (25.1)
≥10	477 (21.6)
Missing	10
***HIV status***	
Negative	1271 (62.4)
Positive	765 (37.6)
Missing	184
***Smear status of index***	
Smear negative	741 (49.2)
Smear positive	766 (50.8)
Missing	713

1 Defined as alcohol consumption four weeks prior to the interview.

A total of 1803 individuals had interpretable results available for both QFT-GIT and TST (figure 1). Of these, 1147 (63.6%) tested positive with QFT-GIT while 725 (40.2%) tested positive with TST (figure 1). In all but two communities infection prevalence as measured by QFT-GIT was higher than that of TST (table 2). Overall, infection prevalence as measured by TST was higher for South African communities (arithmetic mean 50%, range 24–77%) than for the Zambian communities (arithmetic mean 31%, range 7–73%). Results were similar for QFT-GIT (results not shown).

**Table 2:** TB infection prevalence estimates and Cohen's kappa coefficients per community.

Community code	Geography	Urban/rural	1HIV prevalence	Number with both TST & QFT results	QFT-GIT positive (%)	TST positive (10 mm) (%)	Kappa
SA1	Province	Urban	Moderate	109	78	42	0.17
SA 3	Metropole	Urban	High	98	77	77	0.49
SA 6	Province	Rural	Moderate	70	77	27	0.15
Z 4	Lusaka	Urban	High	69	72	41	0.25
SA 5	Metropole	Urban	Moderate	52	71	62	0.28
Z 5	Copperbelt	Urban	High	45	71	13	−0.09
SA 2	Province	Rural	Moderate	87	70	28	0.16
SA 4	Metropole	Urban	High	140	68	61	0.18
Z1	Lusaka	Urban	High	93	67	49	0.36
Z 15	Luapula	Rural	Moderate	24	67	33	0.25
SA 7	Metropole	Urban	High	74	66	76	0.38
Z 11	Luapula	Rural	Moderate	40	65	73	0.02
SA 8	Metropole	Urban	High	124	65	24	−0.01
Z 7	Lusaka	Urban	High	92	61	38	0.19
Z 6	Lusaka	Urban	High	84	61	61	0.48
Z 8	Southern	Urban	High	121	60	42	0.41
Z 3	Copperbelt	Urban	High	48	60	17	0.16
Z 10	Central	Urban	High	68	59	34	0.08
Z 12	Copperbelt	Urban	High	97	59	15	0.19
Z 13	Central	Urban	High	103	50	23	0.16
Z 2	Copperbelt	Urban	High	90	48	28	0.32
Z 16	Southern	Rural	Moderate	18	44	17	0.16
Z 9	Southern	Urban	High	29	38	7	0.22
Z 14	Southern	Rural	Moderate	28	29	11	0.25

Communities arranged from highest to lowest TB infection prevalence estimates as defined by Quantiferon-TB Gold In-Tube (QFT-GIT) test. SA: South African community; Z: Zambian community; TST (Tuberculin skin test). Geography, urban/rural and HIV prevalence as described elsewhere [22], [25].

1 A panel of eight experts critically examined data from ante-natal clinic surveillance, prevention of mother-to-child transmission programmes, voluntary counseling and testing clinics and provincial demographic and health survey data and made an informed decision whether to categorize HIV prevalence as ‘high’ or ‘moderate’ for each community [25].

**Figure 2:**
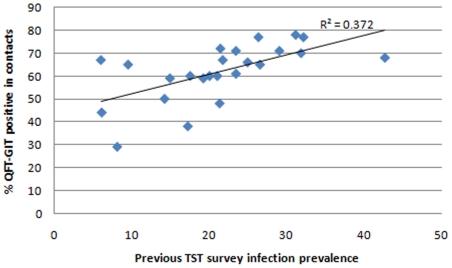
Scatter plot of positive QFT-GIT results in contacts and infection prevalence results from previous TST surveys. Previous TST surveys among children were conducted within the same communities as those of contacts. Infection prevalence in children was defined as TST ≥10 mm.

**Figure 3:**
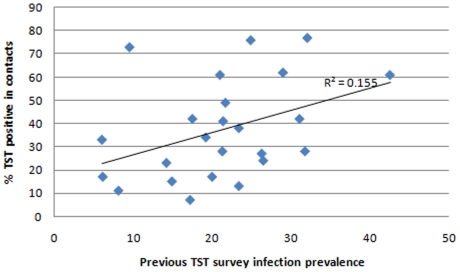
Scatter plot of positive TST results in contacts and infection prevalence results from previous TST surveys. Previous TST surveys among children were conducted within the same communities as those of contacts. Infection prevalence in contacts and children was defined as TST ≥10 mm.

There were 577 (32%) individuals with concordant positive results and 508 (28.2%) had concordant negative results. QFT+/TST− discordant results were more common than QFT−/TST+ results (31.6% vs 8.2%). There was a low level of agreement overall in the 24 communities (% agreement = 60.2%; kappa = 0.24 (arithmetic mean 0.23, range −0.09–0.49)), and in each of the communities (range of kappas: 0–0.49) (table 2). Using different cut-off values for the TST did not improve overall test agreement in the 24 communities (kappa: 0.26, 0.24 and 0.14 for TST cutoffs of 5, 10 and 15 mm respectively). When results were stratified by HIV status, agreement appeared slightly better in HIV positive (% agreement  = 65.1%; kappa 0.322) compared to HIV negative (% agreement =  57.4%; kappa 0.19) contacts. There was a positive correlation (correlation coefficient square, r^2^  = 0.372) between positive QFT-GIT results in contacts and infection prevalence results in children (figures 2), however, this was weaker for the TST (correlation coefficient square, r^2^  = 0.155) (figure 3).

Tables 3 and 4 show risk factors associated with positive QFT-GIT and TST results respectively. Both QFT-GIT and TST results were associated with increasing age (adjusted OR [aOR] for each 10 year increase for QFT-GIT 1.15; 95% CI: 1.06–1.25; p<0.001 for linear trend, and for TST aOR: 1.10; 95% CI 1.01–1.20; p = 0.025 for linear trend). HIV positivity was less common among those with positive results on QFT-GIT (aOR: 0.51; 95% CI: 0.39–0.67; p<0.001) and TST (aOR: 0.61; 95% CI: 0.46–0.82; p = 0.001).

**Table 3:** Univariable and multivariable odds ratios showing risk factors associated with positive QuantiFERON-TB Gold In Tube assay results.

	QFT positive		
	n (row %)	Crude OR (95% CI)	Adjusted OR (95% CI)^1^
Total	1147/1803 (63.6%)		
***Sex***			
Male	333(63.5)	1	1
Female	809 (63.7)	1.08 (0.76–1.25)	0.93 (0.72–1.20)
Missing	5		
***Age group (years)***			
15–24	417 (60.8)	1	1
25–34	276 (61.3)	1.00 (0.74–1.34)	1.00 (0.74–1.34)
35–44	159 (66.5)	1.35 (0.93–1.96)	1.34 (0.92–1.94)
45–54	139 (68.5)	1.49 (1.0–2.22)	1.47 (0.98–2.20)
55–64	87 (75.6)	2.46 (1.43– 4.23)	2.56 (1.47–4.48)
>65	53 (64.6)	1.42 (0.79– 2.53)	1.46 (0.79–2.70)
Missing	16		
***Highest level of education***			
Not attended school	75 (64.7)	1	1
Primary school	378 (67.1)	1.11 (0.68—1.81)	1.4 0 (0.82–2.38)
Secondary school	551 (60.9)	0.79 (0.49–1.28)	0.99 (0.57–1.71)
College or University	111 (67.3)	1.12 (0.62–2.02)	1.40 (0.73–2.69)
Missing	32		
***Smoking habits***			
Never smoked	913 (62.7)	1	1
Ex-smoker	42 (53.9)	0.73 (0.43–1.24)	0.60 (0.34–1.07)
Occasional smoker	43 (70.5)	1.40 (0.73–2.66)	1.13 (0.57–2.25)
Daily smoker	137 (72.9)	1.69 (1.14–2.50)	1.14(0.73–1.77)
Missing	12		
***Alcohol consumption***			
No	844 (62.9)	1	1
Yes	285 (65.4)	1.11 (0.85–1.45)	1.04 (0.78–1.38)
Missing	18		
***Household size (adults)***			
1–3	170 (61.8)	1	1
4–6	415 (62.7)	1.03 (0.73–1.46)	1.30 (0.90–1.87)
7–9	302 (64.4)	1.14 (0.78– 1.67)	1.46 (0.97–2.19)
≥10	258 (65.3)	1.16 (0.77–1.75)	1.68(1.09–2.61)
Missing	2		
***HIV status***			
Negative	728 (69.0)	1	1
Positive	335 (54.6)	0.48 (0.37–0.61)	0.51 (0.39–0.67)
Missing	84		
***Smear status of index***			
Smear negative	373 (60.8)	1	1
Smear positive	426 (67.6)	1.48 (1.09– 2.01)	1.25 (0.90–1.74)
Missing	348		
***Sleeping proximity to index***			
Different house	72(57.6)	1	1
Same house	355 (63.6)	1.31 (0.78–2.18)	1.07 (0.61–1.86)
Same room	36 (56.3)	0.92 (0.42–2.02)	1.19 (0.52–2.72)
Same bed	142 (62.3)	1.26 (0.71–2.23)	1.16 (0.62–2.14)
Unknown	221 (63.1)	1.26 (0.73–2.16)	1.10(0.61–1.99)
Missing	321		

1 Odds ratios-adjusted for sex, age and community using random effects logistic regression.

**Table 4:** Univariable and multivariable odds ratios showing risk factors associated with positive tuberculin skin test results.

	TST ≥10 mm		
	n (row %)	Crude OR (95% CI)	1Adjusted OR (95% CI)
Total	725/1803 (40.2%)		
***Sex***			
Male	203 (38.7)	1	1
Female	520 (40.9)	1.22 (0.92–1.62)	1.18 (0.90 – 1.56)
Missing	2		
***Age group (years)***			
15–24	263 (38.4)	1	1
25–34	177 (39.3)	1.10 (0.79–1.54)	1.05 (0.77–1.45)
35–44	102 (42.7)	1.41 (0.93–2.13)	1.49 (1.00–2.21)
45–54	88 (43.3)	1.34(0.87–2.07)	1.34 (0.89–2.04)
55–64	58 (50.4)	2.01(1.17–3.47)	2.03 (1.19–3.45)
>65	28 (34.1)	1.05(0.55–2.00)	1.12 (0.58–2.17)
Missing	9		
***Highest level of education***			
Not attended school	39 (33.6)	1	1
Primary school	236 (41.9)	1.47 (0.85–2.55)	1.53 (0.88–2.66)
Secondary school	365 (40.3)	1.22 (0.72–2.10)	1.29 (0.72–2.31)
College or University	74 (44.8)	1.49 (0.77–2.90)	1.13 (0.57–2.25)
Missing	11		
***Smoking habits***			
Never smoked	573 (39.3)	1	1
Ex-smoker	32 (41.0)	1.19 (0.64–2.20)	1.09 (0.59–2.02)
Occasional smoker	31 (50.8)	1.51 (0.75–3.02)	1.26 (0.63—2.54)
Daily smoker	85 (45.2)	1.33 (0.87–2.02)	1.10 (0.70–1.73)
Missing	4		
***Alcohol consumption***			
No	544 (40.6)	1	1
Yes	174 (39.9)	0.93 (0.68–1.27)	0.94 (0.69–1.28)
Missing	7		
***Household size (adults)***			
1–3	127 (46.2)	1	1
4–6	261 (39.4)	0.68 (0.44–1.03)	0.77 (0.52–1.13)
7–9	192 (40.9)	0.72 (0.46–1.14)	0.91 (0.59–1.40)
≥10	144 (36.5)	0.57 (0.35–0.94)	0.71 (0.44–1.13)
Missing	1		
***HIV status***			
Negative	465 (44.1)	1	1
Positive	207 (33.8)	0.57 (0.43–0.76)	0.61 (0.46 – 0.82)
Missing	53		
***Smear status of index***			
Smear negative	230 (37.5)	1	1
Smear positive	290 (46.0)	1.65 (1.15–2.36)	1.39 (0.98 – 1.98)
Missing	205		
***Sleeping proximity to index***			
Different house	43 (34.4)	1	1
Same house	202 (36.2)	1.08 (0.62–1.89)	0.76 (0.44–1.30)
Same room	20 (31.2)	0.80 (0.34–1.92)	0.94 (0.41–2.15)
Same bed	91 (39.9)	1.37(0.74–2.55)	0.80 (0.44–1.46)
Unknown	128 (36.6)	1.11 (0.61–2.00)	0.74 (0.41–1.33)
Missing	241		

1 Odds ratios-adjusted for sex, age and community using random effects logistic regression.

There was some evidence of an association between smear positivity of the index and QFT-GIT (aOR: 1.25; 95% CI: 0.90–1.74) and TST (aOR: 1.39; 95% CI: 0.98–1.98) results.

Both QFT-GIT (aOR for household size: 1.04; 95% CI: 1.00–1.09, p = 0.65 for linear trend) and TST (aOR: 0.97; 95% CI: 0.93–1.01, p = 0.005 for linear trend) results were not associated with increasing household size.

Finally, we tested the four specific hypotheses described in the methods. As shown in table 5, we found little evidence to support the hypotheses that positivity in QFT-GIT is more related to recent infection and less affected by HIV than the TST.

**Table 5:** Hypotheses of expected performance of QFT-GIT and TST in our setting and the results obtained.

Prevailing Understanding	Hypothesis	Result
TST is more likely to give false negative results in HIV positives than QFT-GIT.	1. HIV is a risk factor for TST negativity conditional on a QFT-GIT positive result.	Adjusted odds ratio for HIV on TST positivity among QFT-GIT positives = 0.94 (95% CI:0.62-1.40) Wald-test p = 0.75
QFT-GIT positivity is related to recent acquisition of *Mtb* infection whilst TST detects old infections.	2. Age trend is stronger for TST than QFT-GIT because age is as proxy for likelihood of lifetime exposure to *Mtb.*	Wald-test for age'*‘diagnostic test’ interaction parameter in conditional logistic regression; p = 0.94
	3. Stronger association between residence with a smear positive TB case and QFT-GIT positivity than for TST positivity, because smear status is a marker of infectivity and thus of likelihood of recent exposure to *Mtb.*	Wald-test for ‘smear status of index case’*‘diagnostic test’ interaction parameter in conditional logistic regression; p = 0.45
	4. Stronger association between sleeping in same room as index case and QFT-GIT positivity than for TST positivity, because sleeping in the same room is a marker of likelihood of recent exposure to MTB.	Wald-test for ‘sleeping in same room as index’*‘diagnostic test’ interaction parameter in conditional logistic regression; p = 0.76

## Discussion

We conducted a large scale evaluation of the prevalence of LTBI as detected by TST and QFT-GIT among household contacts of tuberculosis patients in 24 high HIV and TB prevalence communities in Zambia and South Africa. Our findings suggest a high prevalence of LTBI among this population. QFT-GIT estimates were higher than those of TST in all but two communities. LTBI prevalence was higher in South African communities compared to the Zambian ones, as in previous findings [22].

LTBI was more common among older individuals and those who were HIV negative, similar to previous studies in this setting [9], [10], [30]. HIV positivity was less common among those with positive results on QFT-GIT and TST. We found little evidence to support the hypothesis that HIV infection was associated with TST negativity among QFT-GIT positive individuals as would have been expected if HIV causes more false negatives with TST than QFT-GIT. Both TST and QFT-GIT are prone to false negatives results among different population groups [9], [30]. In a study done in Zambia, low CD4+ counts in HIV positive TB patients were associated with increases in both indeterminate and false-negative QFT-GIT results [9]. Current evidence suggests that IGRAs perform similarly to the TST at identifying HIV-infected individuals with LTBI [16].

For both QFT-GIT and TST, prevalence of infection was higher in contacts exposed to smear positive index cases compared to smear negative ones, consistent with findings of other studies [31]. Sleeping proximity of the contact to the index case was not associated with either QFT-GIT or TST results. In contrast, a study done in Cape town found an association between *Mtb* contact scores and increasing exposure [19], simliar to findings in the Gambia [21]. Both of these studies had smaller sample sizes compared to our study and were done among HIV negative [19] or few HIV postive contacts [21]. There is growing evidence suggesting a stronger and better defined association between surrogate markers for TB exposure and QFT-GIT results in low TB incidence settings compared to high-TB incidence settings [14], [32], [33], [34] although this is still inconclusive.

Our results suggest that tuberculous infection in adults may often be unrelated to household transmission. It is well recognized that transmission of tuberculosis in high incidence settings occurs not only within households but in the community as well [35], [36] among various social locations [37]. A study in Zimbabwe found that the proportion of ELISpot positive contacts was not different from community controls [31]. In another study done in two communities in Zambia, almost 50% of community controls were QFT-GIT positive [38]. In our study, positive QFT-GIT results in contacts correlated well with infection prevalence results from previous TST surveys, providing further evidence that community transmission seems to play a bigger role in positivity than household exposure. However, in a large study in Colombia, IFN-γ responses to CFP-10 were consistently higher in household contacts of all ages compared to subjects in the source population [34]. Nevertheless, a seven day whole blood culture in-house assay was used, which primarily detects central memory responses. It has been argued that, in settings of high endemicity where a mixture of recent and old infections are commonly found, long term assays are more sensitive than those with shorter culture times [34].

We found little evidence in our matched pair analysis to support the idea that age was more strongly associated with a positive TST result than with a positive QFT-GIT result since we anticipated that TST was more likely to detect evidence of lifetime infection with *Mtb* while QFT-GIT was more likely to detect recent infections. Our results using conditional logistic regression showed that age was associated with positive QFT-GIT and TST results and there was a trend to increased responses with increasing age for both tests suggesting cumulative exposure to *Mtb.* In the study done in Colombia [34], exploration of IFN-γ variations by age revealed a trend to increased responses up to adulthood with CFP, but not with CFP-10, similar to observations in Uganda [39]. However, children were included in both of these studies.

We show a low level of agreement between the tests in all communities, consistent with findings of studies done in high-TB burden settings [19], [31], [40]. As IGRAs are designed to be more specific than TST, perfect agreement is not expected [41]. However, better agreement has been shown when the comparison is done within specific risk groups like HIV positives [42]. Although kappa statistics have been widely used as a measure of agreement between IGRAs and TST, alternative statistical approaches have recently been proposed such as latent class analysis [43] but have yet to gain wider acceptance. Similar to other studies reported from poor-resource settings [44], there were particularly high number of QFT-GIT+/TST- discordant results, in contrast to studies done in settings with low TB incidence [45], [46] where TST+/QFT-GIT- discordance is more common.

Our study had both strengths and limitations. Ours is among the first studies to conduct both TST and QFT-GIT tests using a large sample size which illustrates the realistic implementation of QFT-GIT in a setting with a high burden of TB and HIV. However, as for all studies of this nature we had no gold standard measure of LTBI against which to compare our tests. As such, we were unable to comment directly on the accuracy of either test, but rather to compare the findings of each test in relation to prior beliefs about their properties. It is plausible that our failure to prove our hypotheses may have been due to test limitations typical in such high-TB burden settings. In addition, individuals in these settings may have mixed infections due to multiple *Mtb* exposure.

Our results may have been severely compromised by missing data on some risk factors. Despite efforts to standardize TST training and reading across the two countries, use of different teams may have contributed to inter-reader variability. Although most contacts reported that they had never smoked or taken alcohol, we believe this was due to reporting bias. We had no data on likely exposure to NTM which may provide an alternative reason for false positive results, especially with TST.

Probably the most important characteristic of tests of LTBI is the extent to which they predict subsequent clinical tuberculosis. The data we present here are cross-sectional in nature; however, they come from a larger longitudinal study whose participants have been followed up for later development of active TB.

### Conclusion

QFT-GIT may not be more sensitive than the TST to detect risk factors associated with tuberculous infection**.** Given the lack of strong associations with either TST or QFT-GIT with risk factors generally accepted to be related to household infectivity, these results suggest that tuberculous infection in adults in these communities may often be unrelated to household transmission. We found little evidence to support the hypotheses that positivity in QFT-GIT is more related to recent infection and less affected by HIV than the TST.
